# Role of IGF1R^+^ MSCs in modulating neuroplasticity via CXCR4 cross-interaction

**DOI:** 10.1038/srep32595

**Published:** 2016-09-02

**Authors:** Hsu-Tung Lee, Hao-Teng Chang, Sophie Lee, Chen-Huan Lin, Jia-Rong Fan, Shinn-Zong Lin, Chung Y. Hsu, Chia-Hung Hsieh, Woei-Cherng Shyu

**Affiliations:** 1 Department of Neurosurgery, Taichung Veterans General Hospital, Taichung, 40421, Taiwan; 2 Graduate Institute of Medical Sciences, National Defense Medical Center, Taipei, 114, Taiwan; 3 Graduate Institute of Basic Medical Science, China Medical University, Taichung, 40421, Taiwan; 4 Center for Neuropsychiatry, China Medical University Hospital, Taichung, 40440, Taiwan; 5 Graduate Institute of Life Science, Graduate Institute of Medical Sciences and Department of Neurology, National Defense Medical Center, Taipei, 114, Taiwan; 6 Graduate Institute of Immunology, China Medical University, Taichung, 40440, Taiwan; 7 Graduate Institute of Clinical Medical Science, China Medical University, Taichung, 40421, Taiwan

## Abstract

To guide the use of human mesenchymal stem cells (MSCs) toward clinical applications, identifying pluripotent-like-markers for selecting MSCs that retain potent self-renewal-ability should be addressed. Here, an insulin-like growth factor 1 receptor (IGF1R)–expressing sub-population in human dental pulp MSCs (hDSCs), displayed multipotent properties. IGF1R expression could be maintained in hDSCs when they were cultured in 2% human cord blood serum (hUCS) in contrast to that in 10% fetal calf serum (FCS). Cytokine array showed that hUCS contained higher amount of several growth factors compared to FCS, including IGF-1 and platelet-derived growth factor (PDGF-BB). These cytokines modulates the signaling events in the hDSCs and potentially enhances engraftment upon transplantation. Specifically, a bidirectional cross-talk between IGF1R/IGF1 and CXCR4/SDF-1α signaling pathways in hDSCs, as revealed by interaction of the two receptors and synergistic activation of both signaling pathways. In rat stroke model, animals receiving IGF1R^+^ hDSCs transplantation, interaction between IGF1R and CXCR4 was demonstrated to promote neuroplasticity, therefore improving neurological function through increasing glucose metabolic activity, enhancing angiogenesis and anti-inflammatiory effects. Therefore, PDGF in hUCS-culture system contributed to the maintenance of the expression of IGF1R in hDSCs. Furthermore, implantation of IGF1R^+^ hDSCs exerted enhanced neuroplasticity via integrating inputs from both CXCR4 and IGF1R signaling pathways.

Pluripotency dictates the capacity of stem cells to differentiate into cell types of all three germ layers. Although many investigations focused on identifying growth factors^1^,^2^,^3^ and related cell-surface receptors^4^ for maintaining the pluripotency of embryonic stem cells (ESCs), little is known about the related pluripotent mechanism in MSCs. Several studies suggest that the cell-surface receptors of insulin-like growth factor-1 receptor (IGF1R) are activated when ESCs are exposed to conditions favorable for self-renewal and pleuipotency^4^. As we knew that pluripotent mesenchymal stem cells (MSCs) carry with the same markers as ESCs^5^,^6^,^7^,^8^,^9^, but the role of IGF1R on the regulatory mechanism in MSCs pleuripotency has not been reported^5^,^6^,^7^. In addition, MSCs have unique immunologic characteristics, such as low immunogenicity and immunoregulatory property^10^. IGF1/IGF1R signaling are capable of inducing anti-inflammation effect on the intestine^11^. Immunomodulatory ability of MSCs exerting through IGF1/IGF1R pathway remains to be elusive. IGF1R is highly expressed in the human dental pulp tissue^12^ and pluripotent mesenchymal stem cells could be isolated from dental pulp (human dental pulp stem cells, hDSCs) of 3^rd^ molar with the potential to differentiate into mature cell types including osteoblasts, neurons and hepatocytes^9^,^13^. Thus, we intend to isolate and characterize the mesenchymal stem cells from dental pulp tissue (hDSCs) to comprehensively investigate the regulatory role of IGF1R on hDSCs.

Neuronal SDF-1α and its receptor CXCR4 co-expressing in the adult cerebral cortex and hippocampus suggest that they may also exert neuromodulatory actions in these regions. In addition, cerebral ischemia evoke dramatic changes in neuronal SDF-1 and CXCR4 mRNA expression in non-infarcted areas may suggest that SDF-1/CXCR4 system is important in neuronal plasticity, repair and immunomodulation in the adult brain^14^,^15^. Besides, IGF1R plays a key role in IGF’s neuroprotection and/or promotion of neural regeneration following hypoxia and ischemic injury^16^. Furthermore, development of neural cell lineages is highly dependent on the IGF1/IGF1R signaling pathway^17^. Importantly, whether these two signaling cascade could interact to modulate the cellular physiology. Regarding to the receptors cross-talking, signaling pathway interaction between G proteins coupled receptor (GPCR) and growth factor receptor-tyrosine kinase (RTKs) is well demonstrated in different cellular systems^18^,^19^. Investigation on interactions between different receptor classes is essential for understanding the mechanisms through which cells process multiple signaling inputs simultaneously, yet the potential cross-talk and interaction between IGF1R and CXCR4 signaling pathways on stem cells proliferation and self-renewal has not been explored.

Stem cell therapeutic strategies in regenerative medicine require their pluripotency and self-renewal capacities while tumorogenesis and ethical issues should be avoided in the clinical application. To overcome the ethical issue in using ESCs, the human MSC from bone marrow, adipose tissue^20^ or dental pulp^21^ are under intense investigation for tissue engineering and regenerative medicine because they are relatively easy to be isolated and have the multipotent potential for differentiation. In order to eschew the animal product and prion contamination in the *in vitro* stem cell culture system, we developed a unique culturing cocktail using low percentage of human umbilical cord serum (hUCS) to expand the human dental pulp stem cells (hDSCs). hUCS is endowed with a rich source of different cytokines and has been applied to culture cord blood HSCs^22^, T cells^23^ and BM MSCs^24^. In this study, we intend to investigate the molecular mechanisms that hDSCs incubated in this culture system could retain the long-term self-renewal capacity that is correlated with the expression of IGF1R. Furthermore, we speculated that receptors crosstalk between IGF1R and CXCR4 demonstrated in the metastatic MDA-MB-231 cells^25^ might synergistically stimulate growth signaling in the IGF1R^+^ hDSCs. We also hypothesized that cross-talk between IGF1R and CXCR4 signaling pathways in IGF1R^+^ hDSC might exert autocrine/paracrine induction of an additive survival signal to enhance neurite regeneration and neuroplasticity in hDSC-transplanted stroke model.

## Results

### Quantitative analysis of specific cytokines in hUCS and FCS using cytokines array

**Figure 1:**
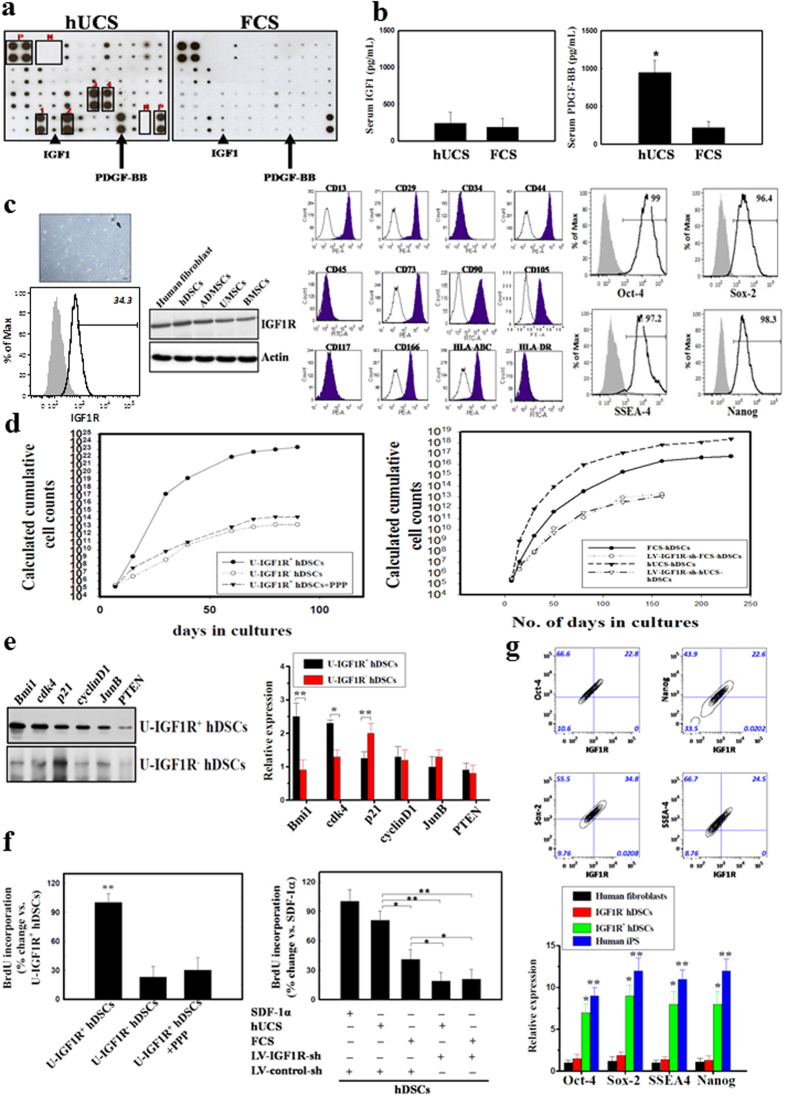
Isolation and clonal expansion of IGF1R-expressed hDSCs. (**a**) In the representative figure of cytokines array (square P = positive control, square N = negative control), five different cytokines such as EGF (square #1), Angiogenin (#2), MIP-1δ (#3), RANTES (#4), and PDGF-BB (arrow) were significantly up-regulated in hUCS compared to FCS. The level of IGF1 (arrow head) was almost the same between hUCS and FCS. (**b**) In ELISA, the concentration of serum PDGF-BB showed significant increase in the hUCS compared to that of FCS, but not found in serum IGF1. (**c**) Fibroblast-like cell morphology (arrow) was found in hDSCs from dental pulp explant (arrow head, left panel). Surface marker of IGF1R and other MSCs’ markers was not only found in hDSCs, but in human fibroblast (positive control), ADMSCs, UMSCs and BMSCs. In flowcytometric analysis, hDSCs carried the surface markers of MSCs and pluripotent markers of Oct-4, Sox-2, Nanog and SSEA-4. (**d**) hDSCs were cultured in 2% hUCS or 10% FCS. In representative graph of the expansion potential profile, U-IGF1R^+^ hDSCs revealed more fast growth rate than that of U-IGF1R^‒^ hDSCs and U-IGF1R^+^ hDSCs+PPP. hUCS-cultured hDSCs revealed rapider division than FCS-cultured hDSCs. In addition, higher cellular proliferation rate was abolished in both hUCS-cultured hDSCs (U-hDSCs) and FCS-cultured hDSCs (F-hDSCs) infected with LV-IGF1R-shRNA. (**e**) Western blot exhibited significant upregulation of Bmi1 and cdk4, as well as downregulation of p21 in U-IGF1R^+^ hDSCs compared to that of U-IGF1R^‒^ hDSCs, but no difference in cyclinD1, JunB and PTEN. (**f**) In BrdU ELISA, U-IGF1R^+^ hDSCs showed significant increaser BrdU uptake compared to that of U-IGF1R^‒^ hDSCs and U-IGF1R^+^ hDSCs+PPP. More, hUCS-cultured hDSCs showed significantly increased BrdU incorporation compared to that of FCS-cultured hDSCs (SDF-1α as positive control), but not in LV-IGF1R-sh-hUCS-hDSCs and LV-IGF1R-sh-FCS-hDSCs. (**g**) Co-expression of IGF1R with self-renewal markers Oct-4, Sox-2, Nanog, and SSEA4 was found on hDSCs by flowcytometry. Furthermore, qRT-PCR analysis of relative expression of Oct-4, Sox-2, Nanog and SSEA4 significantly increased in p5 IGF1R^+^ hDSCs compared to that in IGF1R^–^ hDSCs. Data are expressed as mean ± SEM. *P < 0.05 and **P < 0.01 vs. control, Bar = 40 μm.

In order to evaluate the advantages of hUCS for culturing human stem cells, we first applied an array system (RayBiotech^TM^ Human Cytokine Array III) to identify and compare the specific cytokine(s) in hUCS against FCS. Total of 42 cytokines were examined and the cytokine expression levels in hUCS or FCS were determined by densitometry. We found that the expressions of five different cytokines were significantly higher in hUCS than in FCS (Fig. 1a). These include epidermal growth factor (EGF, 2 fold), angiogenin (ANG, 3 fold), macrophage inflammatory protein (MIP-1δ, 3 fold), regulated on activation- normal T-cell expressed and presumably secreted (RANTES, 2 fold) and platelet-derived growth factor BB (PDGF-BB, 4 folds). In contrast, the level of insulin-like growth factor 1 (IGF1) was almost the same in hUCS and FCS (Fig. 1a). To further quantitate the concentration of specific cytokines, ELISA of PDGF-BB and IGF1 were performed in the hUCS and FCS. Serum concentration of PDGF-BB was substantially higher in hUCS compared to that of FCS, while the level of IGF1 between them was similar (Fig. 1b).

### Clonal IGF1R-expressed hDSCs from dental pulp tissue

To generate hDSC primary cultures, dental pulp tissue was processed into explant culture, and spindle-shaped adherent cells subsequently migrated from the explants. In the immunophenotyping analysis, hDSCs from dental pulp harbored the same markers as those of mesenchymal stem cells (MSCs) (Fig. 1c), consistent with observations of bone marrow MSCs (BMSCs)^26^,^27^. Moreover, hDSCs also express the surface markers of IGF1R, which shows consensus expression in the BMSCs, adipose-derived MSCs (ADMSCs), and umbilical cord-derived MSCs (UMSCs) (Fig. 1c). Pluripotent markers of Oct-4, Sox-2, Nanog and SSEA-4 were present in hDSCs consistently (Figs 1c and S1a). Then, the hDSCs were cultured either in 2% hUCS, or in 10% fetal calf serum (FCS), and passaged as necessary. The hUCS-cultured hDSCs grew exponentially and were extensively expanded (Fig. 1d). To evaluate the proliferation potential, growth kinetic analyses in hUCS-cultured hDSCs revealed that these cells doubled every 22 hours and remained growing for more than 150 days without signs of senescence and spontaneous differentiation. In contrast, FCS-cultured hDSCs showed lower division rate than hUCS-cultured hDSCs (Fig. 1d).

### Maintenance of self-renewal in hDSCs by activating IGF1R signaling

To investigate whether the IGF1R signaling pathway contributes to the regulation of hDSCs self-renewal, receptor knockdown study using lentivirus-mediated shRNA targeting technique was performed. hDSCs infected with Lenti-IGF1R shRNA (LV-IGF1R-sh-hDSCs) revealed a significant reduction in IGF1R proteins 48 hours post-infection compared with non-inhibitory control shRNA (data not shown). The growth kinetic showed that LV-IGFR-sh transduction inhibited rapid cell proliferation in hDSCs cultured in hUCS and FCS (Fig. 1d). Further, significantly fast growth rate was found in the U-IGF1R^+^ hDSCs compared to that of U-IGF1R^‒^ hDSCs and U-IGF1R^+^ hDSCs+PPP (Fig. 1d).

To investigate the molecular mechanisms underlying the enhanced proliferation of U-IGF1R^+^ hDSCs compared to U-IGF1R^‒^ hDSCs, we next examined the expression of several genes previously implicated in self-renewal function. Western blot analysis revealed that U-IGF1R^+^ hDSCs exhibited significantly elevated levels of expression of the polycomb group gene, Bmi1, which previously has been implicated in regulating NSCs self-renewal^28^ (Fig. 1e). In addition, consistent with their increased fraction of cycling cells, U-IGF1R^+^ hDSCs also exhibited significant downregulation of the cell-cycle inhibitor p21CIP1/WAF1 (p21) and increased expression of cyclin dependent kinase 4 (cdk4) (Fig. 1e). Moreover, western blot analysis of cyclin D1, JunB and PTEN revealed no differences in their expression between U-IGF1R^+^ hDSCs and U-IGF1R^‒^ hDSCs (Fig. 1e).

To further evaluate hDSCs proliferation potential, BrdU chemiluminescence ELISA was performed. hUCS-cultured hDSCs showed significant higher BrdU incorporation compared to that of FCS-cultured hDSCs (Fig. 1f). In contrast, LV-IGF1R-sh transduction abolished BrdU incorporation in both hUCS-cultured hDSCs and FCS-cultured hDSCs, indicating IGF1R is essential in hDSC proliferation (Fig. 1f). In addition, higher BrdU uptake was found in the U-IGF1R^+^ hDSCs than that of U-IGF1R^−^ hDSCs and U-IGF1R^+^ hDSCs+PPP (Fig. 1f).

Subsequent flowcytometry of hDSCs revealed that the cells were positive for IGF1R, and co-expressed with pleuripotent markers of Oct-4, Sox-2, Nanog and SSEA4 (Fig. 1g) in 5 independent dental pulp samples. In addition, relative expression of Oct-4, Sox-2, Nanog and SSEA4 by qRT-PCR in p5 IGF1R^+^ hDSCs was higher than that in IGF1R^–^ hDSCs (Fig. 1g). In double immunofluorescent examination, IGF1R co-expressed with Oct-4, Sox-2, Nanog, SSEA4 and CXCR4 in both the hUCS-cultured and FCS-cultured hDSCs (Fig. S1b).

### *In vitro* differentiation of hDSC

To investigate the pluripotent differentiation potential of hUCS- or FCS-cultured hDSCs, fifth to tenth-passaged cells were seeded at a density of 5 × 10^3^ cells/cm^2^ in culture medium. The adipogenic, chondrogenic osteogenic differentiation ability and vascular tubes formation (Fig. S1c) were similar between hUCS-cultured and FCS-cultured hDSCs as previously described^26^. With respect to neural differentiation, some of the cells in the dish exhibited refractile cell body morphology with extended neurite-like structures arranged into a network (Fig. S1c). hDSC-derived neuroglial cells were identified by immunofluorescence with GFAP, MAP-2, O4 and Tuj-1. The percentage of differentiated neuroglial cells was higher in hUCS-cultured hDSCs (GFAP: 15.2 ± 3.1%; MAP-2: 12.1 ± 3.1%; O4: 9.4 ± 2.1%; Tuj-1: 10.2 ± 1.7%) than that in FCS-cultured hDSCs (GFAP: 8.6 ± 2.2%; MAP-2: 7.1 ± 2.7%; O4: 5.8 ± 1.6%; Tuj-1: 6.1 ± 1.5%).

### Bi-directional cross-talk between IGF1R and CXCR4

**Figure 2:**
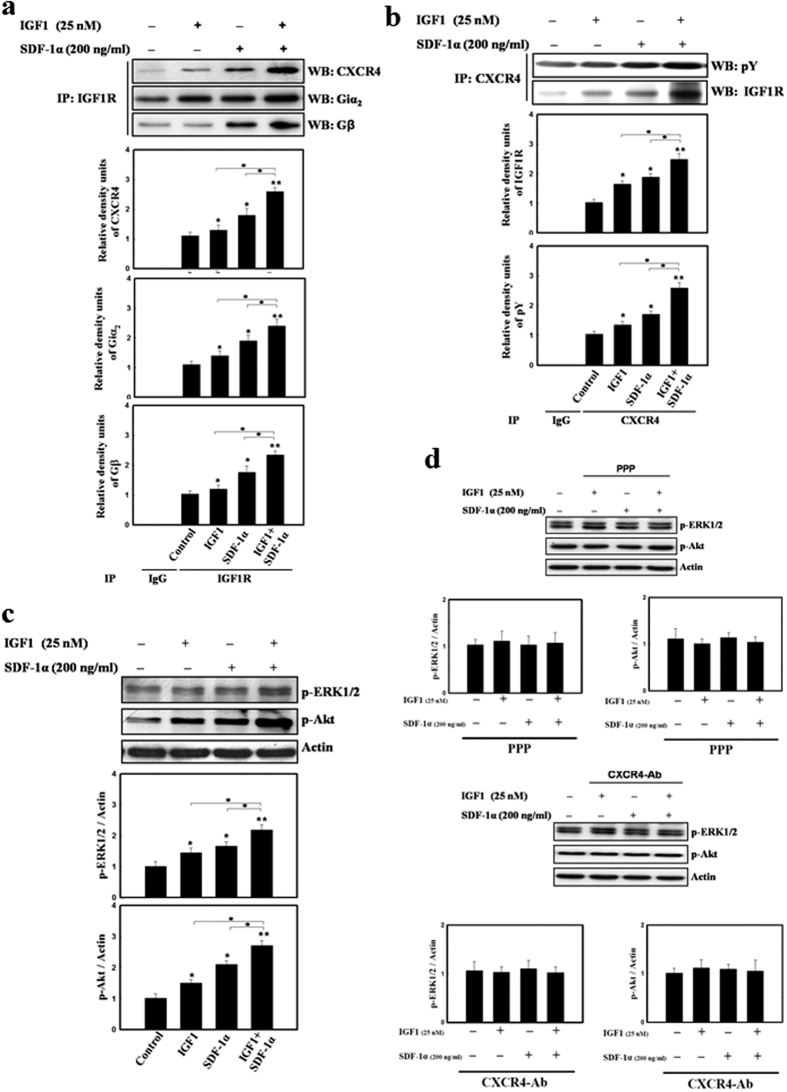
Cross-talk between IGF1R and CXCR4 was co-activated by IGF1 and SDF-1α in hDSCs. (**a**) In immunoprecipitation analysis (IP) with IGF1R, IGF1 (25 nM) or SDF-1α (200 ng/mL) treatment stimulated significantly increase level of CXCR4, active form of Gα_2_ and Gβ in hDSCs by western blot (WB) compared to control (IgG as negative control). Both SDF-1α and IGF1 added in the cultured cells induced an additive expression of the receptors and their major downstream intermediates. (**b**) Under the treatment of IGF1 and/or SDF-1α, IP of CXCR4 following by WB showed significantly increase level of IGF1R and active form of pY in hDSCs compared to control. (**c**) IGF1 or SDF-1α treatment increased phosphorylated-Akt (p-Akt) and phosphorylated-ERK1/2 (p-ERK1/2) in hDSCs. Additive upregulation of p-Akt and p-ERK1/2 were noted in administration of SDF-1α plus IGF1 into hDSCs. (**d**) Pretreatment of inhibitors by PPP (IGF1R inhibitor) or CXCR4 blocking antibody (CXCR4 inhibitor) significantly reduced IGF1 and/or SDF-1α activated p-ERK1/2 and p-Akt in hDSCs. Data are expressed as mean ± SEM. *P < 0.05 and **P < 0.01 vs. control.

To investigate potential receptor interaction between IGF1R, CXCR4 and their downward signaling pathways, the major signaling elements such as phosphotyrosine (pY), Giα_2_, and Gβ were immunoprecipitated from lysed cells treated with IGF1 and/or SDF-1α using an anti-IGF1R, anti-CXCR4, or control IgG, followed by western blot analysis. In the treatment of hDSCs with IGF1 alone, SDF-1α alone or both, immunoprecipitation (IP) of either IGF1R or CXCR4 followed by immunoblotting with CXCR4 or IGF1R showed significant increase in expression of IGF1R, CXCR4, pY, active form of Giα_2_, and Gβ compared to control (without treatment) (Fig. 2a,b). Moreover, IGF1 and SDF-1α co-application to cultured cells caused an additive effect on activating the receptors and their major signaling intermediates compared to either treatment alone. Thus, IP of IGF1R or CXCR4 in the presence of IGF1 and/or SDF-1α stimulation led to co-precipitation of IGF1R, CXCR4, pY, Giα_2_, and Gβ, indicating the existence of a consistent ligand-augmented protein complex in hDSCs (Fig. 2a,b).

We next examined whether IGF1 and SDF-1α had synergistic effect on the downstream signaling molecules. Either IGF1 or SDF-1α treatment resulted in increased levels of phosphorylated Akt (p-Akt) and phosphorylated ERK1/2 (p-ERK1/2) in hDSCs (Fig. 2c). Furthermore, combined treatment of IGF1 and SDF-1α induced an additive increase in p-ERK1/2 and p-Akt (Fig. 2c).

To further verify whether activation of the downstream signaling of ERK1/2 and Akt was through receptor cross-talking between IGF1R and CXCR4, we applied specific receptor inhibitors of IGF1R (PPP) and/or CXCR4 (CXCR4 blocking antibody, CXCR4-Ab) into hDSCs. Intriguingly, PPP inhibited not only IGF1, but also SDF-1α signaling, and led to the decreased levels of p-ERK1/2 and p-Akt in hDSCs. Similarly, pre-treatment of CXCR4-Ab was sufficient to inhibit both SDF-1α and/or IGF1 induced p-ERK1/2 and p-Akt enhancement in hDSCs (Fig. 2d). Together, it suggests that the activation of IGF1R signaling pathway depended on CXCR4, and vice versa.

### Differential regulation of IGF1R and CXCR4 expressions by PDGF-BB and IGF1 in hDSCs

**Figure 3:**
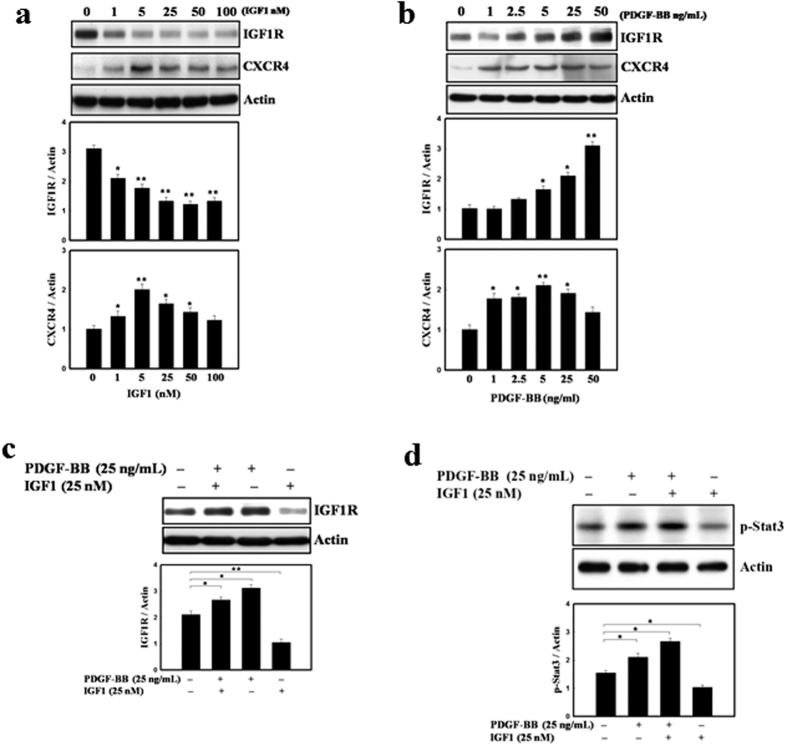
PDGF-BB and/or IGF1 regulated the differential expression of IGF1R and CXCR4. (**a**) In western blot analysis, recombinant IGF1 decreased the expression of IGF1R in hDSCs with a dose-dependent manner. IGF1 treatment in hDSCs increased the expression of CXCR4. (**b**) PDGF-BB protein dose-dependently upregulated the IGF1R and CXCR4 expression. (**c**) In addition, recombinant PDGF-BB reversed IGF1-induced downregulation of IGF1R effectively. (**d**) hDSCs treated with IGF-I and PDGF-BB revealed the same level of Stat3 phosphorylation as the cells treated with PDGF-BB alone. However, even in both growth factors simultaneously adding to cells, IGF-I did not show an additive effect on p-Stat3 activation. Data are expressed as mean ± SEM. *P < 0.05 and **P < 0.01 vs. control.

To investigate whether IGF1R and CXCR4 expression could be regulated differentially by the treatment of IGF1 alone or with PDGF-BB, which is the key ingredient in hUCS, western blot was performed to analyze hDSCs. The result revealed that hDSCs treated with various concentrations of recombinant IGF1 resulted in decreased expression of IGF1R in a dose-dependent manner (Fig. 3a). In contrast, IGF1 administration to hDSCs could upregulate the expression of CXCR4 in a dose-dependent manner (Fig. 3a). In addition, recombinant PDGF-BB dose-dependently increased the IGF1R and CXCR4 expression as demonstrated by western blot analysis (Fig. 3b). Furthermore, IGF1-induced downregulation of IGF1R was effectively inhibited by addition of recombinant PDGF-BB (Fig. 3c).

To further clarify whether PDGF-BB is more dominant than IGF1 in activating proliferation signaling pathway, the level of phosphorylated forms of Stat3 (p-Stat3) were determined in hDSCs treated with IGF1 and/or PDGF-BB under serum-free conditions. The presence of PDGF-BB significantly upregulated the phosphorylation of Stat3 whereas IGF1 alone had no effect. Moreover, when both growth factors were added to cells, IGF1 did not show an additive effect on the level of p-Stat3 (Fig. 3d). IGF1- and PDGF-BB-induced phosphorylation of Stat3 were completely inhibited in the presence of their specific pharmacological inhibitors, AG490 (Jak2/Stat3 inhibitor) (Data not shown). Taken together, these results revealed that PDGF-BB was more effective in activating downstream signaling pathway than IGF1 (Fig. 3d), making hUCS a more superior culture serum than FCS.

### IGF1R^+^ hDSC transplantation improved neurological behavior in stroke rats

**Figure 4:**
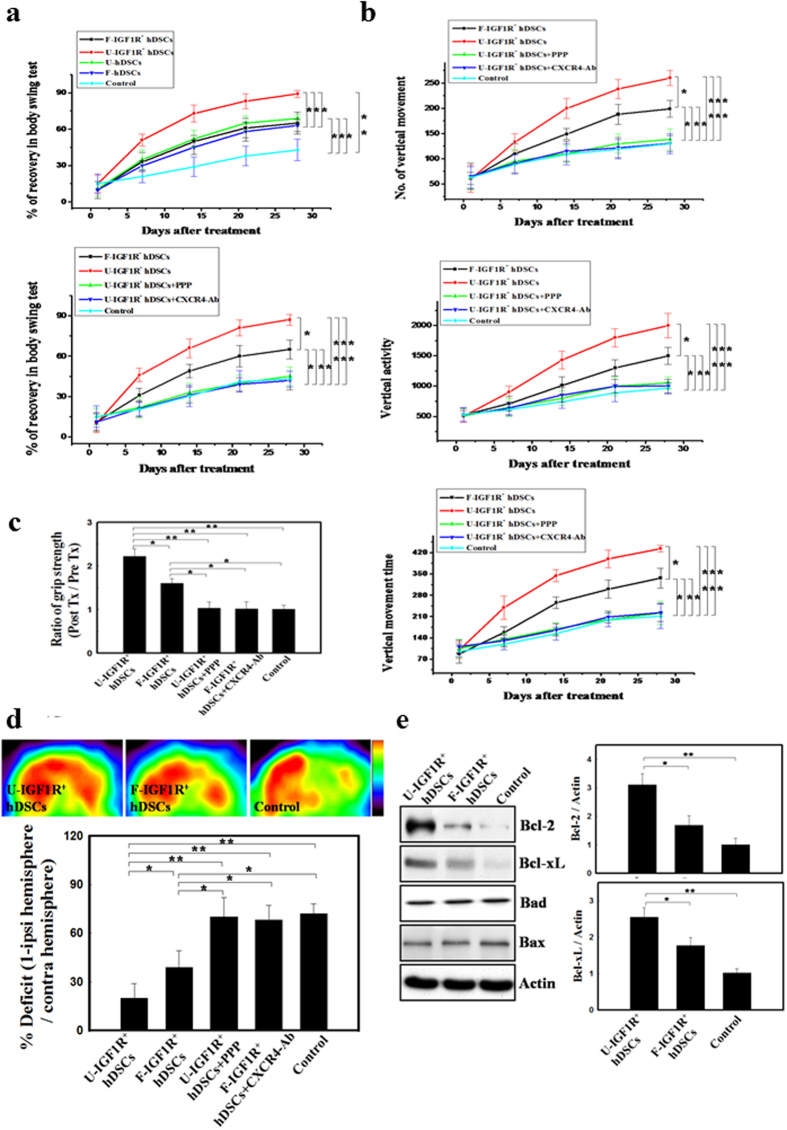
Implantation of IGF1R^+^ hDSCs augmented neurological functional recovery after cerebral ischemia. (**a**) U-IGF1R^+^ hDSC-treated rats showed significant recovery of body asymmetry compared to that of F-IGF1R^+^ hDSCs, U-hDSCs, and F-hDSCs. Better improvement in body swing test was also found in the F-IGF1R^+^ hDSC, U-hDSC, and F-hDSC-treated rats compared to that of control group (upper panel). Experimental rats receiving intracerebral U-IGF1R^+^ hDSC transplantation showed more recovery of body asymmetry than that of F-IGF1R^+^ hDSC-treated and control rats. But the improvement of body asymmetry in U-IGF1R^+^ hDSC-treated rats was abolished by the intraperitoneal injection of specific IGF1R inhibitor (PPP) and CXCR4 blocking antibody (CXCR4-Ab) (lower panel). (**b**) In locomotor activity analysis, U-IGF1R^+^ hDSC-treated rats exhibited significantly increase of vertical activity, vertical movement time, and the number of vertical movements compared to F-IGF1R^+^ hDSC-treated and control populations, but not in U-IGF1R^+^ hDSC+PPP- and U-IGF1R^+^ hDSC+CXCR4-Ab-treated rats. (**c**) In grip strength measurement, experimental rats treated with U-IGF1R^+^ hDSCs revealed a higher ratio of grip strength than that of F-IGF1R^+^ hDSC-treated and the control group. But recovery of grip strength in U-IGF1R^+^ hDSC-treated rats was inhibited by the injection of PPP and CXCR4-Ab. (**d**) In FDG-PET examination, the relative glucose metabolic activity showed significant increase in the U-IGF1R^+^ hDSC-treated group compared to F-IGF1R^+^ hDSC-treated and control. Conversely, injection of PPP or CXCR4-Ab inhibited U-IGF1R^+^ hDSC implantation-induced increase glucose metabolic activity. (**e**) Expression of anti-apoptotic proteins, such as Bcl-2 and Bcl-xL, in the U-IGF1R^+^ hDSC-treated rats’ brain revealed significantly upregulated compared to F-IGF1R^+^ hDSC-treated and control rats. n = 8 in each group, Data are expressed as mean ± SEM. *P < 0.05 and **P < 0.01 and **P < 0.01 vs. control, Bar = 40 μm.

*In vivo* self-renewal and neuroregenerative potentials of IGF1R^+^ hDSC were demonstrated in a stroke model. Three modalities of neurological deficit measurement (body asymmetry, locomotor activity and grip strength) were evaluated before and after stroke in rats. First we subdivided rats into five treatment groups, including U-IGF1R^+^ hDSCs (cultured with hUCS), F-IGF1R^+^ hDSCs (cultured with FCS), U-hDSCs, F-hDSCs and control group to evaluate the behavior recovery in stroke rats. Better recovery was found in U-IGF1R^+^ hDSC-treated rats than that of F-IGF1R^+^ hDSCs, U-hDSCs, and F-hDSCs (Fig. 4a, upper panel). Significant improvement of neurological deficit was also observed in the F-IGF1R^+^ hDSC, U-hDSC, and F-hDSC-treated rats compared to that of control group (Fig. 4a, upper panel). These results suggest that IGF1^+^ hDSCs have superior neuroregenerative potential.

We next evaluated three groups of animal treated with U-IGF1R^+^ hDSCs (cultured with hUCS), F-IGF1R^+^ hDSCs (cultured with FCS) and vehicle-control rats. From 14 to 28 days after treatment, rats receiving intracerebral U-IGF1R^+^ hDSC transplantation exhibited significant improvement in body asymmetry in comparison with F-IGF1R^+^ hDSC-treated and control rats (Fig. 4a). Locomotor activity (vertical activity, vertical movement time, and the number of vertical movements) significantly increased between 14 and 28 days after cerebral ischemia in rats receiving U-IGF1R^+^ hDSC transplantation in comparison with F-IGF1R^+^ hDSC-treated and control populations (Fig. 4b). In addition, rats receiving U-IGF1R^+^ hDSC transplantation showed improved grip strength in comparison with F-IGF1R^+^ hDSC-treated and control rats (Fig. 4c). All modalities of neurological behavior measurement of ischemic rats receiving the specific IGF1R inhibitor (PPP) or CXCR4-Ab (mAb 173) injection in U-IGF1R^+^ hDSC-implanted group showed almost no recovery similar to that of the vehicle control after cerebral ischemia (Fig. 4a–c).

### Enhancement of glucose metabolic activity after stereotaxic U-IGF1R^+^ hDSC transplantation

To verify whether U-IGF1R^+^ hDSC implantation could enhance glucose metabolic activity, ischemic rats were examined by [^18^F]fluoro-2-deoxyglucose positron emission tomography (FDG-PET) using microPET. At 4 weeks after treatment, semiquantitative measurement for the uptake of FDG on the microPET image showed a striking increase in the right cortical region of the U-IGF1R^+^ hDSC-treated group compared to F-IGF1R^+^ hDSC-treated and control rats (Fig. 4d). However, increase in glucose metabolic activity was abolished in the U-IGF1R^+^ hDSC-treated rats injected with PPP or CXCR4-Ab (Fig. 4d).

### Intracerebral transplantation of U-IGF1R^+^ hDSCs increased expression of anti-apoptotic proteins *in vivo*

In order to demonstrate whether the improvement in neurological function was correlated with survival factors after stroke-induced rats receiving U-IGF1R^+^ hDSC transplantation, we examined the expression of anti-apoptotic proteins in the ischemic area using western blot analysis. It showed that significant upregulations of anti-apoptotic proteins of Bcl-2 and Bcl-xL, but not Bax and Bad in U-IGF1R^+^ hDSC-treated rats at 3 days after treatment compared with F-IGF1R^+^ hDSC-treated and control rats (Fig. 4e).

### Stereotaxic U-IGF1R^+^ hDSCs treatment enhanced neural differentiation *in vivo*

**Figure 5:**
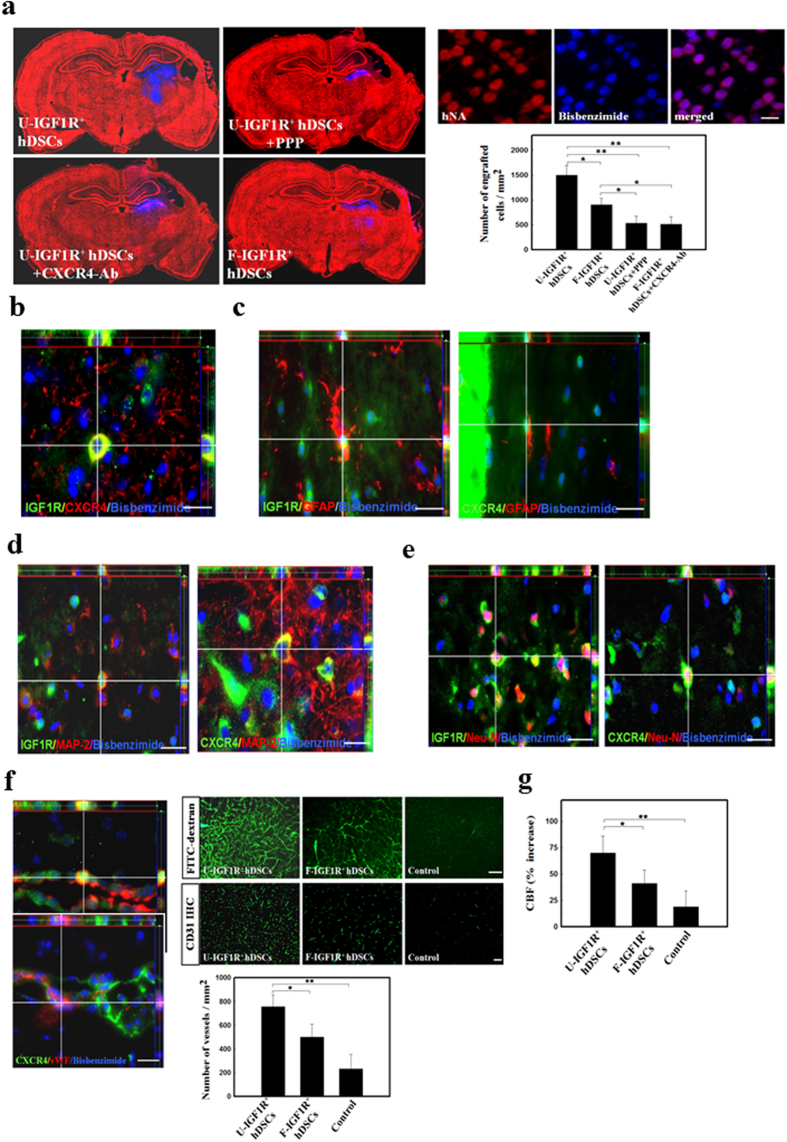
Reciprocal interaction of IGF1R and CXCR4 on U-IGF1R^+^ hDSCs-treated rats promoting neurogenesis and angiogenesis in ischemic brain. (**a**) In colocalization image, bisbenzimide-labeled stem cells co-expressed with human nuclear antigen (hNA). In representative figures of bisbenzimide-labeled stem cells implantation into ischemic brain (red color, PI staining), significantly increased engrafted cell numbers was found in the U-IGF1R^+^ hDSC-treated rats compared to that of U-IGF1R^+^ hDSC-treated rats, but not in the rats injected with PPP and CXCR4-Ab. (**b**) In double immuno-fluorescent study, U-IGF1R^+^ hDSC-treated rats’ brain showed more CXCR4^+^ IGF1R^+^ bisbenzimide^+^ cells in the penumbric area. (**c–e**) Either IGF1R or CXCR4 immunoreactive cells in the penumbra of U-IGF1R^+^ hDSC-treated rats’ brains co-expressed with specific markers for GFAP, MAP-2 and Neu-N on the bisbenzimide-labeled cells. (**f**) By confocal microscopy analysis, U-IGF1R^+^ hDSC-treated rats’ brain revealed that both IGF1R^+^ and CXCR4^+^ in bisbenzimide-labeled cells co-expressed vascular phenotypes (vWF^+^ cells) around the perivascular regions. FITC-dextran perfusion study indicated that U-IGF1R^+^ hDSC-treated rats’ brain have much cerebral microvascular perfusion. Bood vessel density counted by CD31 immunoreactivity in U-IGF1R^+^ hDSC-treated brain showed more neovasculature in the penumbric area than F-IGF1R^+^ hDSCs and control rats. (**g**) In the cerebral blood flow (CBF) measurement by laser doppler flowmetry (LDF), U-IGF1R^+^ hDSC-treated rats’ brain showed more CBF in the middle cerebral artery cortex territory of the ischemic brain than F-IGF1R^+^ hDSC-treated and control rats. n = 8 in each group, Data are expressed as mean ± SEM. *P < 0.05 and **P < 0.01 vs. control, Bar = 40 μm.

To determine whether transplanted U-IGF1R^+^ hDSCs could differentiate into neuroglial cells, double immunofluorescent study using laser scanning confocal microscopy was used to determine the co-localization of cell-type specific markers and bisbenzimide-labeled cell nuclei at 28 days after transplantation. Every hNA^+^ cell colocalized with bisbenzimide-labeled hDSCs indicats the hDSCs as human origin. Transplantation of U-IGF1R^+^ hDSCs showed higher number of engrafted cells in ischemic rats than that of F-IGF1R^+^ hDSC (Fig. 5a). However, the increased cells engraftment in the U-IGF1R^+^ hDSC-treated rats was inhibited by the injection of PPP and CXCR4-Ab (Fig. 5a). In double immuno-fluorescent study, more CXCR4^+^ IGF1R^+^ bisbenzimide^+^ human cells were found in the penumbric area of U-IGF1R^+^ hDSC-treated rats compared to that in F-IGF1R^+^ hDSCs-treated rats (Fig. 5b). Some bisbenzimide-labeled cells that express CXCR4 or IGF1R were colocalized with GFAP, MAP-2 and Neu-N (Fig. 5c–e) in the penumbra of U-IGF1R^+^ hDSC-treated ischemic rat brains. Percentages of bisbenzimide-labeled cells colocalizing with specific markers GFAP, MAP-2 and Neu-N were significantly higher in the U-IGF1R^+^ hDSC-treated rats (≈9.5%, ≈12%, and ≈10%) than that in the F-IGF1R^+^ hDSC-treated rats (≈4%, ≈5%, and ≈4%), suggesting a higher neurogenesis rate in stroke rats receiving U-IGF1R^+^ hDSCs treatment.

### IGF1R^+^ hDSC transplantation promoted angiogenesis *in vivo*

To determine whether U-IGF1R^+^ hDSC implantation could potentiate angiogenesis in the penumbric area of ischemic brain, double immunofluorescent staining, FITC-dextran perfusion studies, and blood vessel density assays were performed on brain slices from U-IGF1R^+^ hDSC-, F-IGF1R^+^ hDSC- and vehicle-control treated rats. The results showed that several bisbenzimide-labeled cells co-expressing IGF1R or CXCR4 were colocalized with cells of vascular phenotype (vWF^+^) in the perivascular and endothelial regions (Fig. 5f) of the ischemic hemispheres in the U-IGF1R^+^ hDSC-treated rats. Visual inspection of brains perfused with FITC-dextran indicated that treatment with U-IGF1R^+^ hDSCs significantly enhanced cerebral microvascular perfusion compared to that of F-IGF1R^+^ hDSCs and control (Fig. 5f). Quantitative measurement of blood vessel density examined by CD31 immunoreactivity showed that ischemic rats treated with U-IGF1R^+^ hDSCs showed more neovasculature in the penumbric area than F-IGF1R^+^ hDSCs and control rats (Fig. 5f).

### U-IGF1R^+^ hDSC implantation facilitated cerebral blood flow (CBF) in the ischemic brain

Increased vessel density, associated with an increased cerebral blood flow (CBF), resulted in efficient delivery of oxygen, nutrients, and enhancing neuronal survival. Therefore, in order to examine CBF in the ischemic brain, blood flow of experimental rats was monitored by laser doppler flowmetry (LDF) under anesthesia after cerebral ischemia. The results revealed a significant increase in CBF of the ischemic cortex in U-IGF1R^+^ hDSC-treated ratscompared with F-IGF1R^+^ hDSC-treated and control rats (Fig. 5g).

### Modulatory role of CXCR4 and IGF1R interaction in U-IGF1R^+^ hDSC-induced neurite regeneration *in vivo* and *in vitro*

**Figure 6:**
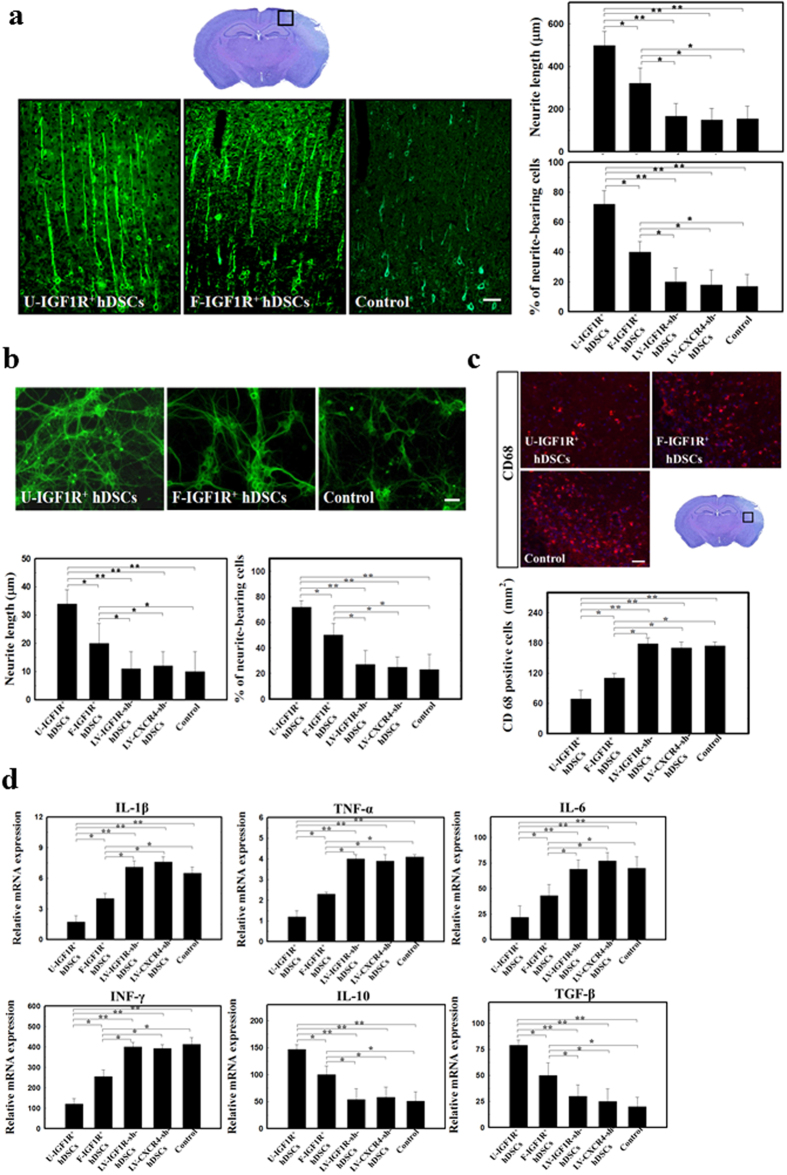
Treatment of hDSCs modulated neurite regeneration by dural interaction of IGF1R and CXCR4 *in vivo* and *in vitro*. (**a**) In representative figures of βIII-tubulin immunoreactivity for stroke rats, U-IGF1R^+^ hDSC implantation revealed significantly longer neurites extended and more neurite-bearing neurons over the penumbral areas than that of F-IGF1R^+^ hDSCs and control rats at 28 days after cerebral ischemia. Experimental rats implanted with LV-IGF1R-sh-hDSCs or LV-CXCR4-sh-hDSCs revealed no improvement of neurite regeneration. (**b**) In representative figures of βIII-tubulin immunoreactivity for PCCs co-cultured with hDSCs under OGD, significantly enhanced neurite length and more neurite-bearing neurons were found in U-IGF1R^+^ hDSCs co-cultured PCCs compared to that of F-IGF1R^+^ hDSCs and controls. Increased neurite length and neurite-bearing neurons were inhibited in PCC co-cultured with LV-IGF1R-sh-hDSCs or LV-CXCR4-sh-hDSCs under OGD. (**c**) Significantly fewer CD68^+^ cells infiltration in the peri-infarct area was noted after U-IGF1R^+^ hDSCs treatment at 3 days after stroke compared to that of F-IGF1R^+^ hDSCs and control rats. Reduction of infiltrated CD68^+^ cells was abolished in LV-IGF1R-sh-hDSCs or LV-CXCR4-sh-hDSCs-treated stroke rats. (**d**) Significant reduction of proinflammatory factors and increased of anti-inflammatory factors in these mRNA expression levels was found in U-IGF1R^+^ hDSC-treated rats compared to the F-IGF1R^+^ hDSCs and control rats. In contrast, decreased proinflammatory factors and increased of anti-inflammatory factors were inhibited in the LV-IGF1R-sh-hDSC- and LV-CXCR4-sh-hDSC-treated rats. n = 8 in each group, Data are expressed as mean ± SEM. *P < 0.05 and **P < 0.01 vs. control, Bar = 40 μm.

In order to demonstrate that interaction of IGF1R^+^ hDSCs and neural tissue stimulated neurite outgrowth *in vivo* and *in vitro*, we quantified neurite regeneration in the stroke rats and an hDSCs/PCCs (primary cortical culture) co-cultured system. Intracerebral U-IGF1R^+^ hDSC transplantation significantly improved axonal regeneration in comparison with that of F-IGF1R^+^ hDSCs and control rats (Fig. 6a, left panel and Fig. S6a). Significantly longer neurites extended over the penumbral areas and striatum of U-IGF1R^+^ hDSC-treated rats than that seen in F-IGF1R^+^ hDSCs and control rats at 28 days after the induced cerebral ischemia (Fig. 6a, left panel and Fig. S6a). Moreover, U-IGF1R^+^ hDSC-treated rats had more neurite-bearing neurons in the penumbral areas and striatum (Fig. 6a, left panel and Fig. S6a). However, increased neurite length and neurite-bearing neurons were abolished in the LV-IGF1R-sh-hDSC- and LV-CXCR4-sh-hDSC-treated rats (Fig. 6a, right panel and Fig. S6a).

To evaluate whether hDSCs co-cultured with PCCs enhanced the neurite regeneration and survival of PCCs, neurite process elongation and the number of survived neurons were measured in PCCs co-cultured with or without hDSCs at 3 days after oxygen glucose deprivation (OGD). Following OGD, neurite length was significantly enhanced and more neurite-bearing neurons were found in U-IGF1R^+^ hDSCs co-cultured PCCs compared to that of F-IGF1R^+^ hDSCs and controls (Fig. 6b, left panel and Fig. S6b). Conversely, PCCs co-cultured with LV-IGF1R-sh-hDSCs and LV-CXCR4-sh-hDSCs under OGD, showed no improvement of neurite regeneration (Fig. 6b, right panel and Fig. S6b).

### Anti-inflammatory effect of U-IGF1R^+^ hDSCs on ischemic brain

To examine whether U-IGF1R^+^ hDSCs implantation suppressed inflammatory response, we performed immunohistochemical analysis for studying the inflammatory cell infiltration and examined the expression of various pro-inflammatory factors on 3 days after U-IGF1R^+^ hDSCs implantation. U-IGF1R^+^ hDSCs treatment showed significantly fewer CD68^+^ cells infiltration at the peri-infarct area at 3 days after stroke than that of F-IGF1R^+^ hDSCs and control rats (Fig. 6c and Fig. S6c). Increased expression of pro-inflammatory cytokines and chemokines were usually observed with many inflammatory cells infiltration in the ischemic brain. Quantitative RT-PCR for assessing expression of various proinflammatory factors (IL-1β, IL-6, TNF-α and INF-γ) and anti-inflammatory factors (IL-10 and TGF-β) at 3 days after treatment revealed significant reduction of proinflammatory factors and increased of anti-inflammatory factors in these mRNA expression levels of U-IGF1R^+^ hDSCs compared to the F-IGF1R^+^ hDSCs and control rats (Fig. 6d). However, decreased CD68^+^ cells infiltration, reduction of proinflammatory factors and increased of anti-inflammatory factors were abolished in the LV-IGF1R-sh-hDSC- and LV-CXCR4-sh-hDSC-treated rats (Fig. 6c,d).

## Discussion

The insulin-like growth factor 1 receptor (IGF-1R) plays a significant role in mitogenic, transforming, and anti-apoptotic signaling^29^. In recent report, Wang *et al*. found that specific IGF1R antibody and siRNA specific for IGF1R reduced the ESCs proliferation and promoted ESCs apoptosis^30^. Thus, IGF1R displays an important function to regulate the ESCs self-renewal and pluripotency^4^,^30^. In addition, IGF1R also plays an essential inter-cellular role in paracrine signaling, which is required for ESCs self-renewal and pluripotency^4^. Therefore, IGF1R seems to be one of newly-defined pluripotent markers, like Oct-4 and Sox-2, in stem cell biology^4^. Although some reports have demonstrated that stem cells from bone marrow or umbilical cord blood contained pluripotent markers such as Oct-4 and/or Sox-2^5^,^31^, we are the first to report that mesenchymal stem cells derived from dental pulp (hDSCs) carried IGF1R, Oct-4 and Sox-2. In agreement with previous study^4^,^30^ we also showed that specific inhibitor of PPP and specific shRNA for IGF1R could reduce the proliferation and self-renewal potential of cultured hDSCs.

With regard to maintain the pluripotency of stem cells such as ESCs, platelet-derived growth factor (PDGF) was able to maintain the survival and proliferation of ESCs in an undifferentiated state for a prolonged period of time^32^. Since PDGF can induce activation of sphingosine kinases (SPKs) to transiently increase the intracellular sphinogosine-1-phosphate (S1P) concentration, administration of S1P combined with PDGF synergistically promote cell proliferation or survival^32^. In a previous report, PDGF stimulated the promoter activity of the IGF1R gene in certain cell types^33^. Higher concentrations of IGF1 inducing downregulation of the IGF1R is a well-known negative feedback mechanism^34^. However, PDGF increased synthesis of the IGF1R in a concentration- and time-dependent manner, and prevented the IGF1-induced downregulation of the IGF1R^34^. In this report, our work on hDSCs not only demonstrated the same findings, but also revealed that PDGF effectively induced phosphorylation of ERK1/2 and PI3K/Akt pathway in contrast to IGF-I, which showed only weak effects. Addition of IGF1 to hDSCs treated with PDGF did not enhance the activation of ERK1/2 and PI3K/Akt pathway. In conclusion, despite previous study has demonstrated that PDGF induced the MSCs proliferation and survival through activation of PDGFRα-related downstream signaling, we are the first to show that hDSCs harboring self-renewal marker of IGF1R might keep their proliferation in an undifferentiated state by PDGF stimulation.

Cellular communication networks represent intercellular receptor transactivation between diverse signaling to allow the translation of complicated environmental condition into appropriate reactions and adaptation^35^. Cross-talk between different cellular signaling is a well-established concept on the stimulus-response conversion system in cell biology^36^. Above all, G-protein-coupled receptors (GPCRs) and receptors tyrosine kinase (RTKs) regulate many downstream signals involved in the MAPK signaling cascade^37^,^38^. The molecular mechanism of GPCRs transactivating RTKs involved the autocrine and/or paracrine release of soluble factors^39^. Second, additive agonist ligands binding to GPCRs could induce a synergistic signaling response. This cellular communication model was so called a dual receptor cross-talk^18^. The mechanism of cross-talk and syngery among two GPCRs induced signaling is mediated by Gβγ exchange between Gαi-coupled and Gαq-coupled receptors^40^. In viewing the sample of GPCRs transactivating RTKs, interaction between TrkA (NGF receptor) and LPA1 (lysophosphatidic acid receptor 1) contributed to NGF signaling in PC12 cells to induce neurite outgrowth^41^. In addition, Delcourt *et al*. have demonstrated that pituitary adenylate cyclase–activating peptide (PACAP) transactivated IGF1R through its type 1 receptor (PAC1) to induce antiapoptotic activity and neuronal survival^42^. Although Akekawatchai *et al*. have found that IGF1 could transactivate CXCR4 signaling in breast cancer cell line independently of SDF-1α to promote cell migration^25^, we have showed that cross-talk between IGF1R and CXCR4 might be transactivated dually by their specific ligands IGF1 and SDF-1α in hDSCs. Furthermore, the final results of these two receptors interaction revealed enhancement of stem cells migration and differentiation, increasing neovasculization and promotion of neurite regeneration. Besides, both IGF1R and CXCR4 transduced the anti-inflammatory signals in different microenvironments^11^,^43^,^44^, which showed consistently in our model after the stem cells treatment.

In regeneration medicine, cultivation of stem cells in FCS might encounter some detrimental problems for clinical application such as prion transmission^45^, immune reaction from contamination with highly immunogenic sialic acid^46^, and anaphylactic reactions^47^. Some commercial available products of complex serum replacement still contained an undefined mixture of animal proteins. To overcome these issues, the use of human serum is strongly recommended for the isolation and expansion of human MSCs. However, studies of MSCs incubated with autologous human serum are still a subject of controversy^48^,^49^,^50^. In the present study, we developed a unique culturing cocktail using lower percentage of human umbilical cord serum (hUCS) for expanding the human DSCs. From the results of cytokine array, hUCS contained significantly higher amounts of growth factors including PDGF in comparison with FCS. Since MSCs expressed a relatively higher level of PDGFRα, higher concentration of PDGF containing in human serum significantly increased the proliferation of MSCs compared to that of FCS^51^. Here, we also found that hDSCs cultivated in hUCS showed significantly higher proliferation rate via upregulation of IGF1R compared to that of FCS. In addition, higher concentration of PDGF in hUCS might prevent the downregulation of IGF1R from the negative effect of IGF1. Implantation of hUCS-cultured hDSCs enhanced the neuroplasticity in the cerebral ischemic rats. Therefore, this study might provide a feasible and safe stem cell culture strategy using hUCS for future clinical application.

Although stem cell transplantation might be an attractive strategy to treat various degenerative disorders, the limited graft survival affects the efficacy of the therapy^52^. Experimental result has demonstrated that only a small percentage of stem cells could survive 1 week after the transplantation^53^. The reason of poor stem cells engraftment into host brain is likely to be affected by the ischemic transplanted environment, where growth factors are lacking, and survival signals from tissue matrix and cell to cell interactions are lost^54^. There are some strategic interventions to overcome these problems to enhance cell survival immediately after transplantation, including the delivery of neuroprotective factors produced by the stem cells^55^, the reduction of toxic substances in the microenvironment^56^, and the replacement of other non-neuronal cells^57^. For examples, genetically engineered cells overexpressing Akt could induced cellular survival after transplantation in the ischemic myocardium and improve the cardiac performance^58^. In addition, genetically modification to induce growth factors and survival protein production from co-implanted fibroblasts, olfactory ensheathing cells (OECs), neural stem cells (NSCs) and embryonic stem cells (ES cells) have been reported to promote host neurological functional recovery after neural injuries. Although these treatment approaches listed above seemed to be very exciting, they were still in preliminary establishment and clinically unfeasible at the current time. Therefore, we need to find simple and applicable strategies in the stem cell treatment for the human stroke. The stem cell populations from primary tissue sources are typically heterogeneous and variable from one isolation to another^59^. In order to select the higher percentage of stem cells harboring the potential of self-renewal and differentiation, several phenotypic markers of stem cells can be applied as the isolation and enrichment methods for clinical transplantation. For examples, previous studies have validated the stem cells enrichment strategies by immuno-magnetic selection methods such as CD34 in hematopoietic stem cells^60^, A2B5 and polysialylated neural cell adhesion molecule (PS-NCAM) in neural progenitor cells^61^, as well as CD49a and CD271 in MSCs^62^,^63^. In the present article, therapeutic strategy using immunosorted-IGF1R^+^ cells by FACS was first applied as a stem cells enrichment tool in hDSCs. We also demonstrated that implantation of IGF1R^+^ hDSCs might be an applicable therapeutic strategy for neurodegenerative diseases.

## Methods

### Human umbilical cord blood serum (hUCS) preparation

Human umbilical cord blood (hUCB, Stemcyte) was harvested in 50-ml Falcon tubes without anticoagulant and processed within 24 hours. Human umbilical cord blood serum (hUCS) was obtained from hUCB by centrifugation (3000 rpm for 10 mins, Beckman) as previously described with modification^64^. Briefly, hUCS was boiled at 56 °C for 30 minutes, passed through a filter (0.22 μm, Stericup, Millipore), and diluted with phosphate-buffered saline (PBS) for further application. All methods and experimental protocols were carried out in accordance with institutional guidelines and regulations, and were approved by an Institutional Committee of Animal and Clinical Research of China Medical University Hospital.

### Cytokine protein array

To investigate the protein expression of cytokines and chemokines in hUCS and fetal calf serum (FCS, Sigma), we applied a cytokine protein array (RayBiotech^TM^) as previously described^65^. The array was performed according to the manufacturer’s instructions. In brief, membranes with immobilized antibodies were incubated overnight with 1 mL of diluted samples (hUCS or FCS containing 200–300 μg protein) at 4 °C. After washing, a mixture of biotin-conjugated antibodies specific for each of the 42 cytokines was reacted on membranes for 3 hours at room temperature. Antigen-bound antibodies were detected with HRP-conjugated streptavidin following by the ECL+ substrate (Amersham), and imaged using an X-ray film (SuperRX; Fuji). Relative densities were semi-quantified using Image J software (National Institutes of Health), and normalized with the average of the six positive controls in each membrane.

### Quantitative measurement of platelet-derived growth factor BB (PDGF-BB) and insulin-like growth factor 1 (IGF1) by ELISA

The total amount of PDGF-BB and IGF1 in hUCS and FCS was measured using a DuoSet IC ELISA Kit (R&D Systems) according to the manufacturer’s instructions. Optical density was measured using a spectrophotometer (Molecular Devices Corporation). The standard curves and levels of PDGF-BB and IGF1 in hUCS and FCS were obtained using the program SoftmaxTM (Molecular Devices Corp.).

### Human dental pulp stem cells (hDSCs) preparation, immunophenotyping and cell sorting

Normal human second or third molars were collected in sterile boxes containing Hanks’ balanced salt solution (HBSS; Gibco/BRL 14185-052) from childrens (5–10 years of age) at the Dental Clinics. The Institutional Review Board (IRB) committee of China Medical University and Hospital, Taichung, Taiwan approved the collection guidelines. Written informed consent was obtained from all donors. All experimental protocols and methods related to the hDSCs were carried out in accordance with the Institutional guidelines, and were approved by an Institutional Committee of Animal and Clinical Research of China Medical University Hospital. The collected human dental tissues were washed three times with Ca^2+^ and Mg^2+^-free PBS (DPBS, Life Technology). Tooth surfaces were cut around the cementum-enamel junction by using sterilized dental fissure burs to reveal the pulp chamber. The dental pulp tissues were gently separated from the crown and root and then digested in a solution of 0.1% collagenase type I (Sigma Aldrich) for 1 h at 37 °C. Single-cell suspensions were obtained by passing the cells through a 70-mm strainer (Falcon) and incubated for 14–18 hours at 37 °C in a 95% air/5% CO_2_ humidified atmosphere. The explants then were plated and expanded in fibronectin (5 ng/mL) coated flasks with medium: DMEM-LG containing either 2% hUCS or 10% FCS supplemented with 20 ng/mL EGF and 20 ng/mL bFGF (R&D), and antibiotics (100 U penicillin/1000 U Streptomycin) (Gibco-BRL) at 37 °C in a 95% air/5% CO_2_ humidified atmosphere. They were left undisturbed for 5–7 days to allow migration of the cells from the explants. The cellular morphology became homogenously spindle-shape in cultures after 4–8 passages, and the specific cell surface molecules were characterized by flowcytometric analysis. The cells were detached with 2 mM EDTA in PBS, washed with PBS containing 2% BSA and 0.1% sodium azide (Sigma Aldrich), and incubated with respective antibodies [conjugated with fluorescein isothiocyanate (FITC) or phycoerythrin (PE)], including CD13, CD29, CD34, CD44, CD45, CD73, CD90, CD105, CD117, CD166, HLA-ABC, HLA-DR (BD PharMingen), Oct-4 (Santa Cruz Biotechnology), Sox-2 (Chemicon), SSEA-4 (R&D System), Nanog (R&D System), CXCR4 (R&D System) and IGF1R (Santa Cruz Biotechnology). Thereafter, the cells were analyzed using a FACSTAR^+^ flow cytometer (Becton Dickinson).

In cells sorting, IGF1R^+^ hDSCs (>95% IGF1R) were purified using a FACSTAR^+^ flow-cytometer (Becton Dickinson) according to previously published methods^66^,^67^. The viability of the sorted cells remained about 96%, as confirmed by Trypan blue exclusion.

### Immunocytochemical analysis

For immunocytochemistry, cell cultures from hDSCs were washed with PBS and fixed for 30 minutes at room temperature in 41% paraformaldehyde. After washing with PBS, the fixed cultured cells were treated for 30 minutes with blocking solution (10 g/L BSA, 0.03% Triton X-100, and 4% serum in PBS). Cells were incubated overnight at 4 °C with an antibody against Sox-2 (1: 200, Millipore), IGF1R (1:200, Abcam), IGF1 (1:200, Abcam), CXCR4 (1:100, R&D System), Oct-4 (1:50, SC-8628, Santa Cruz Biotechnology), GFAP (1:200, Chemicon), MAP-2 (1:200, Chemicon), Doublecortin (Dcx, 1:200, Chemicon) and Tuj-1 (1:200, Chemicon) conjugated with FITC or Cy-3 (1:500, Jackson Immunoresearch). Finally, some of the preparation was lightly counterstained with DAPI, and then mounted. The preparations were analyzed with a Carl Zeiss LSM510 laser-scanning confocal microscope.

### Bromodeoxyuridine (BrdU) proliferation assay

Proliferation of hDSCs was tested by measuring incorporation of BrdU using BrdU chemiluminescence immunoassay kits purchased from Roche (Germany) and further confirmed by standard Trypan blue cell counting. After a 4- to 6-h starvation (medium without supplements), hDSCs were incubated in medium containing 10% FCS or 2% hUCS with supplement or SDF-1α (100 ng/mL, positive control) for 2 days and pulse loaded with 10 μM BrdU for 12 h as previous described^68^. hDSCs were then incubated with anti-BrdU-peroxidase for 90 min and further developed with substrate solution for 3 min. The plates were read with an Lmax microplate luminometer (Molecular Devices). Then the results were analyzed and presented as percentage (%) increase *vs*. control.

### Quantitative RT-PCR (qRT-PCR)

Total RNA was extract from human fibroblast (C-013-5C, Life Technologies), human iPS cells (SBI) and hDSCs by the single-step method of isolation using Trizol (Invitrogen), and then was reverse transcribed with random hexanucleotides using the SuperScript III First-Strand Synthesis System (Invitrogen) as prescribed described^69^. PCR was performed with JumpStart Taq DNA polymerase (Sigma). In quantitative PCR, 50 ng of total RNA was typically used as template in 20 ml SYBR green PCR reactions (40 cycles of 15 s, 95 °C/60 s, 60 °C) on Applied Biosystems 7300 that additionally contained 0.375 mM of each primer^70^,^71^ and 10 ml of SYBR green PCR mix (ABI).

### *In vitro* differentiation assay

Adipogenic differentiation was induced according to the method described previously^72^. In brief, confluent monolayer cultures of hDSCs were grown in adipogenic differentiation medium, consisting of DMEM-high glucose (HG, Sigma), 10% FCS or 2% hUCS, 100 U/ml penicillin, and 100 mg/ml streptomycin which was supplemented with 100 mM insulin, 500 mM 3-isobutyl-1-methylxanthine, 1 mM dexamethasone, and 100 mM indomethacin. Cells maintained in hDSCs medium served as a negative control. Medium changes were performed three times a week. To assess adipogenic differentiation, cells were stained for 10 min at room temperature with 0.3% oil red O, as an indicator for intracellular lipid accumulation, and counterstained with haematoxylin^72^.

Osteogenic differentiation was conducted in confluent monolayer cultures of hDSCs grown in DMEM-high glucose containing 10% FCS or 2% hUCS, 100 U/ml penicillin, 100 mg/ml streptomycin, 50 mg/ml L-ascorbic acid 2-phosphate, 10 mM b-glycerophosphate, and 100 nM dexamethasone^73^. Cells maintained in hDSCs medium were served as negative controls. The medium was changed three times per week. Osteogenesis was determined using alizarin red S staining (1%) to detect calcium mineralisation^74^.

Chondrogenic differentiation of hDSCs was induced using a high-density pellet cell culture system^75^. The cells were washed in serum-free chondrogenic differentiation medium consisting of DMEM-high glucose, 100 U/ml penicillin, 100 mg/ml streptomycin, 50 mg/ml L-ascorbic acid 2-phosphate, 40 mg/ml proline, 100 mg/ml sodium pyruvate, 100 nM dexamethasone, and ITS-plus (10 mg/ml bovine insulin, 5.5 mg/ml transferrin, 5 mg/ml sodium selenite, 4.7 mg/ml linoleic acid, and 0.5 mg/ml bovine serum albumin). Aliquots of 250,000 cells were resuspended in chondrogenic differentiation medium, centrifuged at 250 × g and 10 ng/ml TGF-ß1 (R&D Systems) were added. Pellets maintained in chondrogenic differentiation medium without addition of TGF-β1 were served as negative controls. Medium changes were performed twice a week. Chondrogenic differentiation of pellet cultures was confirmed histologically after alcian blue staining for sulphated proteoglycans^75^. In addition, Endothelial cell differentiation was obtained to become vascular tubes formation by culturing hDSCs in EBM (Cambrex) on 24-well plates precoated with Matrigel (300 AL/well; Becton Dickinson) and vascular endothelial growth factor (VEGF, 10 ng/ml, Sigma) for 2–3 days as described previously^76^.

For differentiation of neural cells, hDSCs were incubated with DMEM using a three-step method as previously described^77^. Briefly, in the neural induction step, cells were plated at low density on 6-well plates containing fibronectin and were specified by exposing cells to DMEM-high glucose (HG, Sigma Aldrich), 2% hUCS, 10 ng/mL bFGF (R&D System) for 24 hours. Then, in the neural commitment step, the cells were exposed to DMEM-HG, 1 mM β-mercaptoethanol (βME, Sigma Aldrich), and 10 ng/mL NT-3 for 2 days. Finally, in the neural differentiation step, they were treated with NT-3 (10 ng/mL, R&D System), NGF (10 ng/mL, R&D System) and BDNF (50 ng/mL, R&D System) in DMEM-HG for 3 to 7 days. Immunocytochemical staining for neuron specific class III β-tubulin (Tuj-1, 1:300; Chemicon, USA), MAP-2 (1:200; Chemicon, USA), O4 (1:300; Millipore) and GFAP (1:300; Chemicon) was used to assess the capacity of hDSCs for neuronal differentiation.

### Gene silencing with RNA interference

For shRNA experiments, cells were plated into 10 cm^2^ culture dishes at a density of 5 × 10^4^/cm^2^ and infected with the Lenti-IGF1R shRNA (LV-IGF1R-sh, sc-29358-V, Santa Cruz Biotechnology), Lenti-CXCR4 shRNA (LV-CXCR4-sh, sc-35421-V, Santa Cruz Biotechnology) and Lenti-control shRNA (LV-control-sh, Santa Cruz Biotechnology) under manufacture’s instruction for 48 hours. Cell proliferation and protein expression of IGF1R or CXCR4, were examined for Lenti-IGF1R-shRNA infected-hDSCs (LV-IGF1R-sh-hDSCs) or Lenti-CXCR4- shRNA infected-hDSCs (LV-CXCR4-sh-hDSCs).

### Total protein extraction and western blotting

hDSCs were allowed to grow until 80% confluence. Before the addition of stimuli, cultures of hDSCs in serum-free medium were washed in phosphate-buffered saline (PBS) and then incubated in the presence or absence of recombinant rat PDGF-BB (Invitrogen), recombinant human IGF1 (Invitrogen) and recombinant human SDF-1α (R&D System) for 6 hours. Pharmacological inhibitors AG490 (p-Jak2/Stat3 inhibitor), LY294002 (p-Akt inhibitor), PD98059 (p-ERK1/2 inhibitor), diluted in dimethylsulfoxide (DMSO) were purchased from Sigma. All cultures received the same amount of solvent were served as control. After treatment, cells were rinsed in cold PBS and immediately used for protein extraction.

Western blot analysis was performed as previously described^78^ hDSCs, BMSCs^79^,, ADMSCs, UMSCs^26^ and human fibroblast (C-013-5C, Life Technologies) were resuspended in a buffer containing 320 mM sucrose, 5 mM HEPES, 1 μg/mL leupeptin, 1 μg/mL aprotinin. Lysates were centrifuged at 13,000 *g* for 15 min. The resulting pellet was lysed in sample buffer (62.5 mM Tris-HCl, 10% glycerol, 2% SDS, 0.1% bromophenol blue, and 50 mM DTT) and subjected to SDS-polyacrylamide gel (4–12%) electrophoresis. The gel was then transferred to a Hybond-P nylon membrane. This membrane was followed by incubation with appropriately diluted antibodies to IGF1R (1:200, Santa Cruz Biotechnology), CXCR4 (1:100, R&D System), p-ERK1/2, ERK1/2 (1:200-1:300; Cell Signaling), p-Akt and Akt (1:100, Calbiochem), Bmi-1 (1:300, Chemicon), cdk4 (1:100, Santa Cruz), cyclinD1 (1:250, Chemicon), JunB (1:200, Chemicon), PTEN (1:200, Chemicon), p-Stat3 and Stat3 (1:200, Santa Cruz Biotechnology), Bcl-2 (dilution 1:200; Santa Cruz Biotechnology), Bcl-xL (dilution 1:200; Transduction Laboratories), Bax (dilution 1:200; Santa Cruz Biotechnology), Bad (dilution 1:200; Transduction Laboratories) and β-Actin (dilution 1:2000, Santa Cruz Biotechnology). Membrane blocking, primary and secondary antibody incubations, and chemiluminescence reactions were conducted for each antibody individually according to the manufacturer’s protocol. The intensity of each band was measured using a Kodak Digital Science 1D Image Analysis System (Eastman Kodak).

### Co-immunoprecipitation analysis

The immunoprecipitation experimental procedures were as previously described^54^. Cultures were treated with recombinant human IGF1 (Invitrogen) at 300 ng/mL^80^ and/or recombinant human SDF-1α (R&D System) at 100 nM^81^. First, hDSCs were collected and lysed in lysis buffer (50 mM Tris-HCl, pH 7.5, 1% NP-40, 150 mM NaCl, 0.5% sodium deoxycholate, and protease inhibitor). The cell lysate (300 μg) was incubated with protein A/G-agarose beads at 4 °C for 6 hours. Then, antibodies against IGF1R (Santa Cruz Biotechnology), CXCR4 (R&D System), phosphotyrosine (pY) (p-Tyr-100, Cell Signaling), Giα_2_ (T-19, Santa Cruz Biotechnology) and Gβ (M-14, Santa Cruz Biotechnology), or control antibody IgG (anti-hemagglutinin clone F-7, Santa Cruz Biotechnology) were added and reacted for 6 hours at 4 °C. These immunocomplexes were incubated on protein A/G-agarose beads at 4 °C overnight. After three washes with lysis buffer, the immunocomplexes were then examined by western blot with anti-IGF1R, anti-phosphotyrosine, anti-CXCR4 antibodies, anti- Giα_2_ and Gβ, or anti-control IgG.

### Animal brain ischemia/reperfusion model

Adult male Sprague-Dawley rats (weight 250–300 g) were used in this study. Animals were subjected to three-vessel ligation. All surgical procedures, animals’ experimental protocols and methods were carried out in accordance with the Institutional guidelines and were approved by the Institutional Committee of Animal and Clinical Research of China Medical University, Taichung, Taiwan. The rats were anesthetized with chloral hydrate (0.4 g/kg, ip). Ligation of the right middle cerebral artery (MCA) and bilateral common carotids (CCAs) was performed by methods described previously, with slight modifications^82^. The bilateral CCAs were clamped with non-traumatic arterial clips. Using a surgical microscope, a 2 × 2 mm craniotomy was drilled where the zygoma fuses to the squamosal bone. The right MCA was ligated with l0-0 nylon suture. Cortical blood flow was measured continuously with a laser Doppler flowmeter (PF-5010, Periflux system, Sweden) in anesthetized animals. A burr hole (l-mm diameter) was made in the right frontoparietal region to allow placement of photodetectors. A probe (0.45 mm in diameter) was stereotaxically placed in the cortex (l.3 mm posterior, 2.8 mm lateral to the bregma, and l.0 mm below the dura). After 90 minutes of ischemia, the suture on the MCA and arterial clips on CCAs were removed to allow reperfusion. Core body temperature was monitored with a thermistor probe and maintained at 37 °C with a heating pad during anesthesia. After recovery from anesthesia, body temperature was maintained at 37 °C with a heat lamp^83^.

### Intracerebral transplantation of IGF1R^+^ hDSCs

For cell labeling, immunosorted IGF1R^+^ cells by FACSTAR^+^ flow-cytometer were cultured in DMEM (Gibco, USA) with 2% hUCS (U-IGF1R^+^ hDSCs) or 10% FCS (F-IGF1R^+^ hDSCs) and antibiotics, at 37 °C in a humidified atmosphere of 5% CO_2_/95% air. Prior to transplantation, the cells were labeled using 1 μg/mL bis-benzimide (Hoechst 33342; Sigma) for 1 hours at 37 °C as previously described^54^. Labeled cells were then collected and washed in PBS three times. IGF1R^+^ hDSCs (>95% IGF1R) were prepared in a 10 μL PBS through a 26-gauge Hamilton syringes. One week after brain ischemia, adult male Sprague-Dawley rats (weight > 300 g) were anesthetized with chloral hydrate (0.4 g/kg, ip) and injected stereotaxically with approximately 1 × 10^6^ cells into 3 cortical areas adjacent to the right MCA, 3.0 to 5.0 mm below the dura. The control animals were administered PBS only. The approximate coordinates for these sites were l.0 to 2.0 mm anterior to the bregma and 3.5 to 4.0 mm lateral to the midline, 0.5 to l.5 mm posterior to the bregma and 4.0 to 4.5 mm lateral to the midline, and 3.0 to 4.0 mm posterior to the bregma and 4.5 to 5.0 mm lateral to the midline. The needle was retained in place for 5 minutes after each injection and a piece of bone wax was applied to the skull defects to prevent leakage of the injected solution. Because of the immunosuppressive characteristics of mesenchymal stem cells^84^, rat hosts did not receive any immunosuppressive medication. For blocking experiment, the CXCR4 neutralization was administered to the IGF1R^+^ hDSCs-treated rats by intraperitoneal injection of CXCR4 blocking antibody (CXCR4-Ab, mAb 173, R&D System) twice weekly for two weeks as described previously^85^. Moreover, IGF1R^+^ hDSCs implanted rats were treated intraperitoneally with specific inhibitor of IGF1R (PPP, 20 mg/kg/day, Santa Cruz Biotechnology) for three days as previously described^86^.

### Neurological behavioral assessment

Behavioral assessments were performed 5 days before cerebral ischemia, and 1, 7, 14 and 28 days after cell transplantation. The tests measured body asymmetry, locomotor activity and grip strength. The baseline-test scores were recorded in order to normalize those taken after cerebral ischemia. The elevated body swing test was used to assess body asymmetry after MCA ligation and evaluated quantitatively as previous described^54^. Initially, animals were examined for lateral movement, on their bodies being suspended by their tails 10 cm above the cage floor. The frequency of initial head swing contra-lateral to the ischemic side was counted in twenty continuous tests and was normalized to the baseline score. Locomotor activity measurement was subjected to VersaMax Animal activity monitoring (Accuscan Instruments, Inc., Columbus, OH) for about 2 hours for behavioral recording^54^. This instrument contained 16 horizontal and 8 vertical infrared sensors. The vertical sensors were situated 10 cm from the floor of the chamber. Motor activity was counted as the number of beams broken by a rat’s movement in the chamber. Three parameters of vertical items over 2 hours were calculated: (i) vertical activity (ii) vertical time (iii) number of vertical movements. Furthermore, grip strength was analyzed using Grip Strength Meter (TSE-Systems, Germany) as previously described, with modification^87^. In brief, the grip strength ratio of each forelimb was measured separately and was calculated as the ratio between the mean strength out of 20 pulls of the side contralateral to the ischemia and the ipsilateral side. In addition, the ratio of grip strength post-treament and pre-treatment was also calculated and the changes were presented as a percentage of the pre-treatment value.

### [^18^F]fluoro-2-deoxyglucose positron emission tomography (FDG-PET) examination

To assess the metabolic activity and synaptic density of brain tissue, experimental rats were examined using microPET scanning of [^18^F]fluoro-2-deoxyglucose (FDG) to measure relative metabolic activity under the protocol previously described^88^. In brief, ^18^F was produced by the ^18^O(p, n)^18^F nuclear reaction in a cyclotron at China Medical University and Hospital, Taichung, Taiwan, and ^18^F-FDG was synthesized as previously described^89^ with an automated ^18^F-FDG synthesis system (Nihon Kokan, Tokyo, Japan). Data were collected with a high-resolution small-animal PET (microPET, Rodent R4, Concorde Microsystems Inc., Knoxville, TN). The system parameters have been described previously by Carmichael *et al*.^90^. After 4 week of each treatment, animals were anesthetized with chloral hydrate (0.4 g/kg, ip), fixed in a customized stereotactic head holder and positioned in the microPET scanner. Then the animals were given an intravenous bolus injection of ^18^F-FDG (200–250 μCi/rat) dissolved in 0.5 ml of saline. Data acquisition began at the same time and continued for 60 min using a 3-D acquisition protocol. The image data acquired from microPET were displayed and analyzed by IDL ver. 5.5 (Research Systems, Colorado, USA) and ASIPro ver.3.2 (Concorde Microsystems Inc., Knoxville) software. FDG-PET images were reconstructed using a posterior-based 3-dimentional iterative algorithm^91^ and overlaid on MR templates to confirm anatomical location^92^. Coronal sections for striatal and cortical measurements represented brain areas between 0 and +1 mm from bregma, while for thalamic measurements between −2 and −3 mm from bregma, as estimated by visual inspection of the unlesioned side. The relative metabolic activity in regions of interest (ROIs) of the striatum was expressed as a percentage deficit as previously described with modification^90^.

### Immunohistochemical assessement of brain tissue

Animals were anesthetized with chloral hydrate (0.4 g/kg, ip) and their brains fixed by transcardial perfusion with saline, followed by perfusion and immersion with 4% paraformaldehyde. Finally, the brain samples were dehydrated in 30% sucrose. After brains were frozen on dry ice, a series of adjacent 6-μm-thick sections were cut in the coronal plane with a cryostat, stained with H&E and observed by light microscopy (Nikon, E600). Blue color fluorescence (from bis-benzimide) of brain sections was detected directly by fluorescence microscopy (Carl Zeiss, Axiovert 200 M).

### Laser-scanning confocal microscopy for immunofluorescence co-localization analysis

In order to demonstrate the differentiation potential of transplanted cells, the expression of cell type-specific markers in bizbenzimide-labeled IGF1R^+^ hDSCs were identified by immunofluorescent analysis of each brain section. Each coronal slides was first stained with primary IGF1R antibody (1:1000; mouse monoclonal, Santa Cruz), human nuclear antigen (hNA, 1:100, Chemicon) and secondary antibody conjugated with FITC (1:500; goat anti-mouse IgG, Jackson Immunoresearch), followed by treatment with specific antibodies reacted to secondary antibody conjugated with Cy3 (1:500; goat anti-rabbit IgG, Jackson Immunoresearch), such as GFAP (1:400; rabbit polyclonal, Sigma), vWF (1:400; rabbit polyclonal, Sigma), Neu-N (1:200; rabbit polyclonal, Santa Cruz), microtubule-associated protein 2 (MAP-2, 1:200; rabbit polyclonal, Santa Cruz), CD68 (1:200, Millipore) and CXCR4 (1:100, R&D System). The tissue sections were analyzed with a Carl Zeiss LSM510 laser-scanning confocal microscope. FITC (green) and Cy3 (red) fluorochromes on the immunofluorescence labeled slides were excited by laser beam at 488 nm and 543 nm, respectively. IGF1R, hNA labeled with Cy3 (red) or FITC (green) fluorochromes, and cell-type-specific markers, Neu-N, MAP-2, vWF, GFAP, CD68 and CXCR4, labeled with Cy3 (red) or FITC (green) fluorochromes were double immunostained in order to demonstrate their co-localization in one cell under laser-scanning confocal microscopy. The total number of cells co-stained with bis-benzimide and cell-type specific markers were measured as previously described^93^.

### Evaluation of angiogenesis

Cerebral microcirculation was analyzed by administering the fluorescent plasma marker (FITC-dextran, Sigma) intravenously to rats and observing them with fluorescent microscopy (Carl Zeiss, Axiovert 200M), as previously described^94^. In addition, to quantify the cerebral blood vessel density experimental rats were anesthetized with chloral hydrate and perfused with 4% paraformaldehyde. Histological sections (6 μm) were stained with specific antibody to CD-31 (1:100, BD Pharmingen), and conjugated with Cy-3 (1:500, Jackson Immunoresearch). The number of blood vessels density was determined as previously described^95^.

### Measurement of cerebral blood flow (CBF)

At one week after treatment, experimental rats were positioned in a stereotaxic frame and baseline local cortical blood flow (bCBF) was monitored after cerebral ischemia with a laser doppler flowmeter (LDF monitor, Moor Instrutments, Axminster, U.K.) in anesthetized state (chloral hydrate) as previously described^78^. In brief, CBF values were calculated as percentage increase compared to the bCBF.

### Assessment of neurite regeneration *in vivo* and *in vitro*

Brain tissue samples and PCCs were immunostained to measure neurite outgrowth as described earlier^96^. Briefly, brain tissue samples and PCCs were fixed and immunostained with specific antibody against βIII-tubulin (1:400; Chemicon). For quantification analysis, neurons with processes greater than twice the cell body diameter were counted as neurite-bearing cells. The length of the longest neurite in each neuron was measured from digitized images and quantified using imaging analysis software (SigmaScan).

### *In vitro* primary cortical culture (PCCs) preparation

Primary cortical cultures (PCCs) were prepared from the cerebral cortex of gestation day 17 embryos from C57BL/6 mice as described previously^97^. PCCs were maintained under serum-free conditions in neurobasal medium (Invitrogen), supplemented with B-27 supplement (2%; Invitrogen), glutamine (0.5 mM; Sigma Aldrich), glutamate (25 mM; Sigma Aldrich), penicillin (100 U/ml) and streptomycin (100 mg/ml; Invitrogen). At 4 days *in vitro*, half of the medium was removed and replaced with fresh medium without glutamate, as indicated by the manufacturer. The cultures were maintained in a humidified incubator at 37 °C with 5% CO_2_. At 7 days *in vitro*, PCCs were used for experimentation.

### Co-cultures of hDSCs with PCCs, and oxygen glucose deprivation (OGD)

For direct co-cultures, hDSCs (5 × 10^4^) were added to PCCs at a density of 100,000 cells in each well of a 6-well tissue culture plate. Co-cultures were maintained in naurobasal medium with B27. The medium was changed once a week. One milliliter of fresh medium was added to each well every 2 days for a total of 8 days. For oxygen glucose deprivation (OGD) treatment, the co-cultured cells were incubated with glucose-free Earle’s balanced salt solution, placed in a hypoxic chamber (Bug Box, Ruskinn Technology) for 4 hours and continuously flushed with 95% N_2_ and 5% CO_2_ at 37 °C to maintain a gas phase PO_2_ of <1 mmHg (OM-14 oxygen monitor; SensorMedics). Control cells were incubated in glucose-free Earle’s balanced salt solution in a normoxic incubator (95% air and 5% CO_2_) for the same period. OGD was terminated by switching back to normal culture conditions. After OGD treatment, the cells were returned to a 37 °C normoxic incubator for different time periods (30 min, 1 hr, 4 hr, 12 hr, 24 hr and 48 hr) of reoxygenation.

### RNA isolation and real time RT-PCR

Total RNA was isolated from ischemic brain with RNA TRIzol (Invitrogen) and performed reverse transcription with the High Capacity cDNA Archive Kit (Applied Biosystems) as well as real-time PCR with SYBR-Green assays (Applied Biosystems) on a GeneAmp 5700 SDS from Applied Biosystems as described previously^98^. Taqman primer/probe sets for various cytokines and housekeeping genes were designed using PRIMER EXPRESS software (Applied Biosystems). Real Time PCR was run using a total of 5 ng template cDNA for each sample. The fast PCR protocol consisted of an initial denaturing step at 95 °C for 20 s. Next, samples were run at 94 °C (denaturation) for 3 s, 60 °C (annealing) for 30 s for 25 cycles. Gene expression levels of TNF-α, IL-1β, IL-6, interferon-γ (INF-γ), IL-10, TGF-β were quantified in the border zone of infarct as described previously^99^. We ran all assays in duplicate. Relative gene expression was calculated using the Ct method with normalization to GAPDH or 18S.

### Statistical analysis

All measurements in this study were performed in a blinded design. Results were expressed as mean ± SEM. The behavioral scores have been evaluated and adjusted by normal distribution. Two-way ANOVA with appropriate post hoc Newman-Keuls testing was used to evaluate differences between different groups with different treatments. A value of *P* < 0.05 was taken as significant.

## Additional Information

**How to cite this article**: Lee, H.-T. *et al*. Role of IGF1R^+^ MSCs in modulating neuroplasticity via CXCR4 cross-interaction. *Sci. Rep.* **6**, 32595; doi: 10.1038/srep32595 (2016).

## Supplementary Material

Supplementary Information
