# Supplementary material for: Role of IGF1R+ MSCs in modulating neuroplasticity via CXCR4 cross-interaction
Source: Sci Rep. 2016 Sep 2;6:32595. doi: 10.1038/srep32595 (PMC5009335; doi:10.1038/srep32595)
Supplement: Supplementary Information [file srep32595-s1.doc]

**Supplementary File**

**Role of IGF1R^+^ MSCs in modulating neuroplasticity via CXCR4 cross-interaction**

Hsu-Tung Lee^1^, **Hao-Teng Chang**^2^, Sophie Lee^3^, Chen-Huan Lin^3^, Jia-Rong Fan^3^, Shinn-Zong Lin^3,4^, Chung Y. Hsu^5^, Chia-Hung Hsieh^2^**^†^** and Woei-Cherng Shyu^3,4^**^†^**

1 Department of Neurosurgery, Taichung Veterans General Hospital, Taichung, Taiwan 40421; and Graduate Institute of Medical Sciences, National Defense Medical Center, Taipei, Taiwan

2 Graduate Institute of Basic Medical Science, China Medical University, Taichung, Taiwan 40421

3 Center for Neuropsychiatry, China Medical University Hospital, Taichung, Taiwan, 40440; Graduate Institute of Life Science, National Defense Medical Center, Taipei, Taiwan.

4 Graduate Institute of Immunology, China Medical University, Taichung, Taiwan, 40440

5 Graduate Institute of Clinical Medical Science, China Medical University, Taichung, Taiwan 40421

**^†^**To whom correspondence should be addressed: C.H.H ([chhsiehcmu@mail.cmu.edu.tw](mailto:chhsiehcmu@mail.cmu.edu.tw)) and W.C.S. ([shyu9423@gmail.com](mailto:shyu9423@gmail.com))

**Supplementary results**

**Maintenance of self-renewal in hDSCs by activating IGF1R signaling.** In double immunofluorescent examination, IGF1R co-expressed with Oct-4, Sox-2, Nanog, SSEA4 and CXCR4 in both the hUCS-cultured and FCS-cultured hDSCs. (Fig S1b).

**In vitro differentiation of hDSC.** To investigate the pluripotent differentiation potential of hUCS- or FCS-cultured hDSCs, fifth to tenth-passaged cells were seeded at a density of 5 x 10^3^ cells/cm^2^ in culture medium. The adipogenic, chondrogenic osteogenic differentiation ability and vascular tubes formation (Fig S1c) were similar between hUCS-cultured and FCS-cultured hDSCs as previously described [^24^](#_ENREF_24). With respect to neural differentiation, some of the cells in the dish exhibited refractile cell body morphology with extended neurite-like structures arranged into a network (Fig S1c). hDSC-derived neuroglial cells were identified by immunofluorescence with GFAP, MAP-2, O4 and Tuj-1. The percentage of differentiated neuroglial cells was higher in hUCS-cultured hDSCs (GFAP: 15.2±3.1%; MAP-2: 12.1±3.1%; O4: 9.4±2.1%; Tuj-1: 10.2±1.7%) than that in FCS-cultured hDSCs (GFAP: 8.6±2.2%; MAP-2: 7.1±2.7%; O4: 5.8±1.6%; Tuj-1: 6.1±1.5%).

**Supplementary Figure legends**

**Figure S1** **Isolation and clonal expansion of IGF1R-expressed hDSCs cultured in different serum environment. (a).** By western blot, IGF1 (25 nM) administration induced the Oct-4 expression in hDSCs, ADMSCs, UMSCs and BMSCs (control = BMSCs without IGF1 treatment). (**b**) Using double immunofluorescent study, IGF1R could also co-stained with Oct-4, Sox-2, SSEA4, Nanog and CXCR4 on both hUCS-cultured and FCS-cultured hDSCs. **(c).** Representative figures for adipogenic differentiation (Oil red O stain), chondrogenic differentiation (Alcion blue stain), osteogenic differentiation (Alizarin red S stain) and vascular tubes formation (upper panel). In neural differentiation of hDSCs, refractile cell body morphologies in part of the cells exhibited with extended neurite-like structures arranged into a network (phase-contrast, left lower panel). The hDSC-derived neuroglial cells were immunoreactive for mature neural markers of GFAP, MAP-2, O4 and Tuj-1 (right lower panel).

**a**


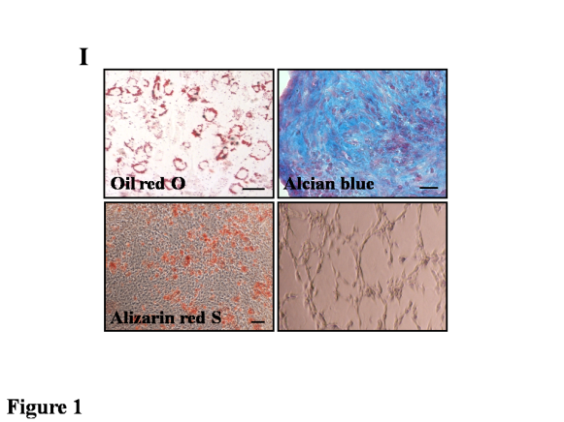

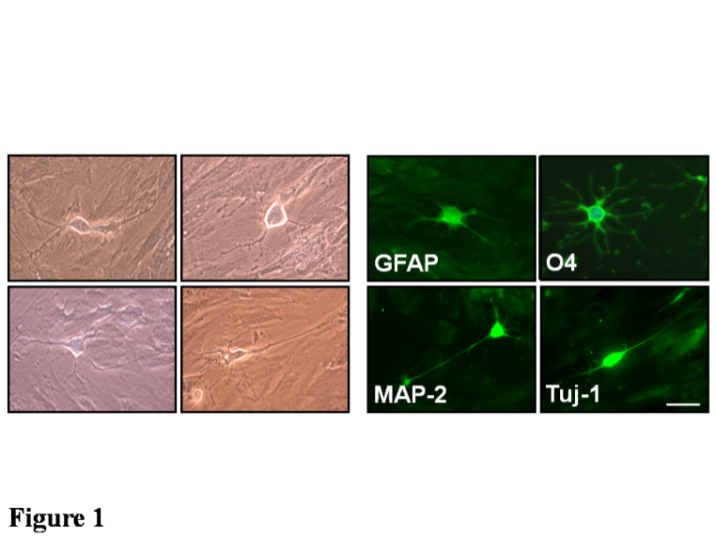


**b**

**c**


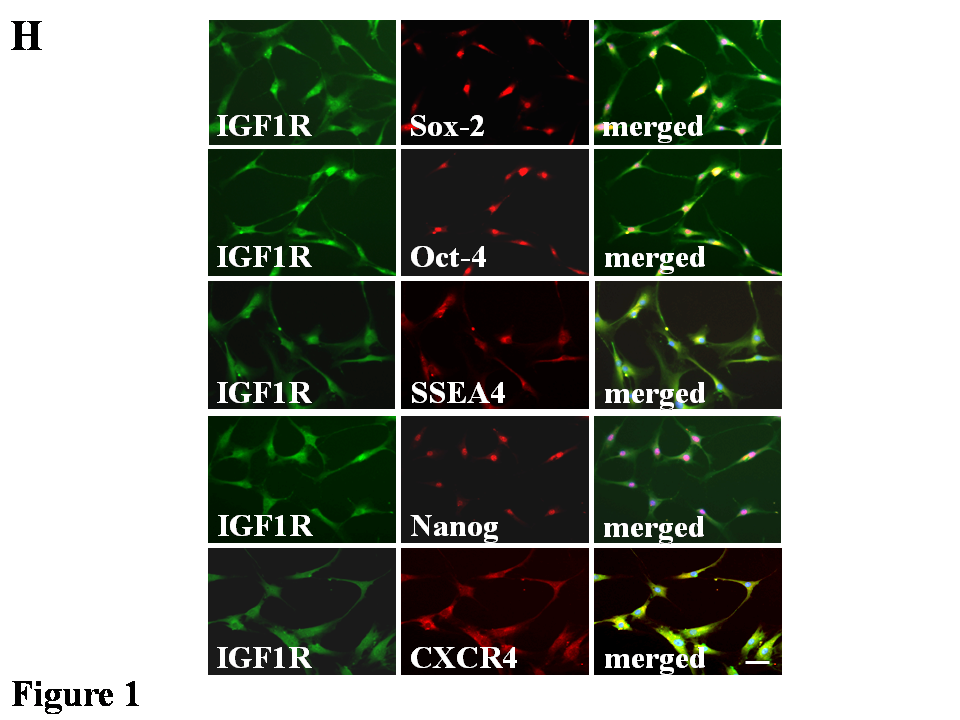


**Figure S6** (**a-b**) neurite regeneration (βIII-tubulin immunoreactivity) *in vivo* and *in vitro.* (**c**) CD68^+^ cells.

**a**

**b**

**c**
